# A non-replicative antibiotic resistance-free DNA vaccine delivered by the intranasal route protects against canine leishmaniasis

**DOI:** 10.3389/fimmu.2023.1213193

**Published:** 2023-09-18

**Authors:** Ana Alonso, Pedro José Alcolea, Jaime Larraga, María Paz Peris, Adriana Esteban, Alberto Cortés, Silvia Ruiz-García, Juan Antonio Castillo, Vicente Larraga

**Affiliations:** ^1^ Laboratory of Molecular Parasitology and Vaccines, Department of Cellular and Molecular Biology, Centro de Investigaciones Biológicas Margarita Salas, Consejo Superior de Investigaciones Científicas (CIBMS-CSIC), Madrid, Spain; ^2^ Laboratory of Parasitology, Department of Animal Pathology, Faculty of Veterinary Science, University of Zaragoza, Zaragoza, Spain

**Keywords:** *Leishmania infantum*, canine leishmaniasis, LACK, third-generation vaccines, pPAL, protection, clinical signs, parasite burden

## Abstract

*Leishmania infantum* is the etiological agent of zoonotic visceral leishmaniasis (ZVL). The disease is endemic in Central and South America, Central and South East Asia, and the Mediterranean basin. Dogs are the main reservoir, with an estimated prevalence of approximately 2.5 million dogs in Southern Europe. Current treatments cause side effects, disease recurrence, and drug resistance. Therefore, the development of vaccines against canine leishmaniasis is necessary. We have generated a DNA vaccine based on the non-replicative antibiotic resistance marker-free plasmid vector pPAL that contains the encoding gene for the *L. infantum* activated protein kinase C receptor analog (LACK). Homologous pPAL-LACK prime-boost intranasal administration confers efficacious protection in Beagle dogs with a reduction of clinical signs and a statistically significant reduction of the parasite burden in the bone marrow of more than 90% of dogs after experimental infection with highly infective promastigotes. This DNA vaccine elicits a robust cellular immune response skewed towards the Th1 profile.

## Introduction

Leishmaniasis is a vector-borne infectious disease caused by intracellular parasites of the *Leishmania* genus (Kinetoplastida: Trypanosomatidae). The parasite is transmitted by phlebotomine sand flies (Diptera: Psychodidae). Human visceral leishmaniasis (VL), caused by *L.donovani*, is fatal without treatment and leads to approximately 50,000 annual deaths worldwide (1). The parasite has a digenetic and dimorphic life cycle where differentiation and development of the promastigote stage occurs in the vector gut and the amastigote stage in phagocytic cells of the mammalian host. Dogs are the main reservoir of *Leishmania infantum* (syn. *L. chagasi*), which is responsible for zoonotic visceral leishmaniasis (ZVL), a public health problem. Asymptomatic dogs play an important role in disease transmission (1). Dogs with leishmaniasis display variable cutaneous and visceral clinical signs simultaneously. The clinical profiles range from asymptomatic to severe and include weight loss, cutaneous lesions, conjunctivitis, and lymphadenopathy (1–3). ZVL is endemic in Central and South America, Central and South East Asia, and the Mediterranean basin. *L. infantum*-HIV co-infections in humans are a serious clinical problem and have been frequently described (4–8). A “One Health” approach would contribute to efficient surveillance and control of ZVL and VL. Climate change is causing the northward spread of the disease, reaching countries previously free of this parasite (9).

Canine leishmaniasis is a life-threatening chronic disease. Available treatments such as pentavalent antimonials, which have been used for over fifty years, lead to side effects, drug resistance, and relapse (10). Vaccines are essential and one of the best methods for the effective control of reservoirs, including asymptomatic animals (11).

The immune response against *Leishmania* spp. is well understood. Type 1 helper T-cell response (Th1) is related to protection, whereas type 2 helper T-cell response (Th2) is associated with susceptibility (12). Interferon-γ (IFN-γ) production is associated with a Th1 cell response and Interleukin 10 (IL-10) secretion is related to Th2 proliferation. This allows for the prediction of the prevalent T cell subset and the fate of the disease (13). However, the Th1/Th2 dichotomy is not as polarized in canine and human leishmaniasis as in the murine model (14–22). As stated, canine leishmaniasis is a chronic disease with marked variability in its clinical signs. The parasite persists in the bone marrow (23) and the most reliable parameter to follow infection is parasite burden. For this reason, the main parameter to measure vaccine efficacy is the parasite load in this tissue.

DNA vaccines have been used in veterinary and human medicine against pathogens to a limited extent, even though they induce specific protective responses. At present, only a vaccine against hemorrhagic fever in salmonids (24) and a vaccine against SARS-CoV-2 infection in humans (25) are available, and preclinical studies with a pPAL-based vaccine have been performed in the mouse model (26). It has been shown that they induce a robust cellular and humoral immune response. DNA vaccines are thermotolerant. This is an advantage for distribution to dog shelters, veterinary clinics, hunting dogs, stray dogs, and developing countries. In addition, multivalent vaccines with two or more protective antigens to improve protection levels can be easily developed and DNA vaccine manufacturing can be easily scaled up.

Herein, we describe the preclinical development of a DNA vaccine against canine leishmaniasis delivered by the intranasal route following a homologous prime/boost immunization regimen. This vaccine is based on the pPAL plasmid, a non-replicative mammalian expression plasmid vector free of antibiotic resistance markers (15, 16) that induces immunomodulation based on the Th1 T-cell response (27, 28). The vaccine contains several CpG islands as immune system activators and the encoding gene of the protective antigen, *L. infantum* Activated Protein Kinase C Receptor Analogue (LACK). The LACK antigen, located in the particulate fraction of the cytoplasm (29), protects BALB/c mice against *L. major* (30, 31), and mice and hamsters against *L. infantum* (32–34). It also protects Beagle dogs against experimental *L. infantum* infection when administered as a DNA vaccine by the subcutaneous route (18, 19, 35). This study aims to evaluate the efficacy of the pPAL-LACK DNA vaccine against canine leishmaniasis in the Beagle dog model under controlled preclinical conditions.

## Materials and methods

### Parasites and antigens

The *L. infantum* isolate, MCAN/ES/MON1/Z001, was retrieved from a bone marrow explant of a naturally infected dog and cultured at 26°C in NNN (Novy–Nicolle–McNeal) medium. This isolate holds genotype A and zymodeme MON-1 (Drs. M. Jiménez and R. Molina, Spanish National *Leishmania* Reference Laboratory, personal communication) according to ITS- (36) and CPB-based (37) genotyping and zymodeme determination (38, 39). After initial *in vitro* cultivation and genotyping, promastigotes were transferred to complete medium (CM) composed of RPMI 1640 supplemented with 2 mM glutamine (Gibco BRL, Waltham, MA), 10% heat-inactivated (56°C, 1 h) fetal bovine serum (Cambrex, East Rutherford, NJ), and 100 μg streptomycin / 100 IU penicillin/mL (Gibco BRL Waltham, MA). Stationary phase (day 7) promastigotes were obtained for challenge four passages after bone marrow explant cultivation. Crude *L. infantum* antigen (CLA) was obtained from 8x10^8^ cells/mL stationary phase promastigote suspensions in PBS applying three freeze-thaw cycles (-20°C/room temperature).

The recombinant LACK protein was obtained using a pRSETB-LACK BL21 *E. coli* clone following a described heterologous expression and purification procedure (14). The purified LACK protein fractions were dialyzed three times against PBS (30 min each), and finally against 0.1% SDS in PBS for 16 h. CLA and LACK antigens were quantified by the Bradford method.

### pPAL-LACK plasmid vaccine

Obtention of the pPAL-LACK third-generation vaccine has been described (15, 16, 40). The pPAL-LACK construct does not contain the SV40 replicon. This component and the SV40 promoter were removed from the pCI-neo-LACK plasmid (16). The antibiotic resistance genes *nptII* and *bla* were replaced by the *E. coli fabI* selection marker (16). Hence, the pPAL-LACK construct is not replicative and lacks antibiotic resistance markers. The plasmid has been recommended for use as a vaccine by the CVMP of the European Medicines Agency under the name of Neoleish^®^ (EMA/CVMP/858971/2022). Large endotoxin-free laboratory-scale preparations were obtained with the EndoFree Plasmid Giga Kit (Qiagen, Maryland, MD). LACK antigen gene expression in HEK293 cells transfected with pPAL-LACK was evaluated and confirmed as described (16).

### Animals, ethics statements, plasmid inoculation, and infectious challenge

**Figure 1:**
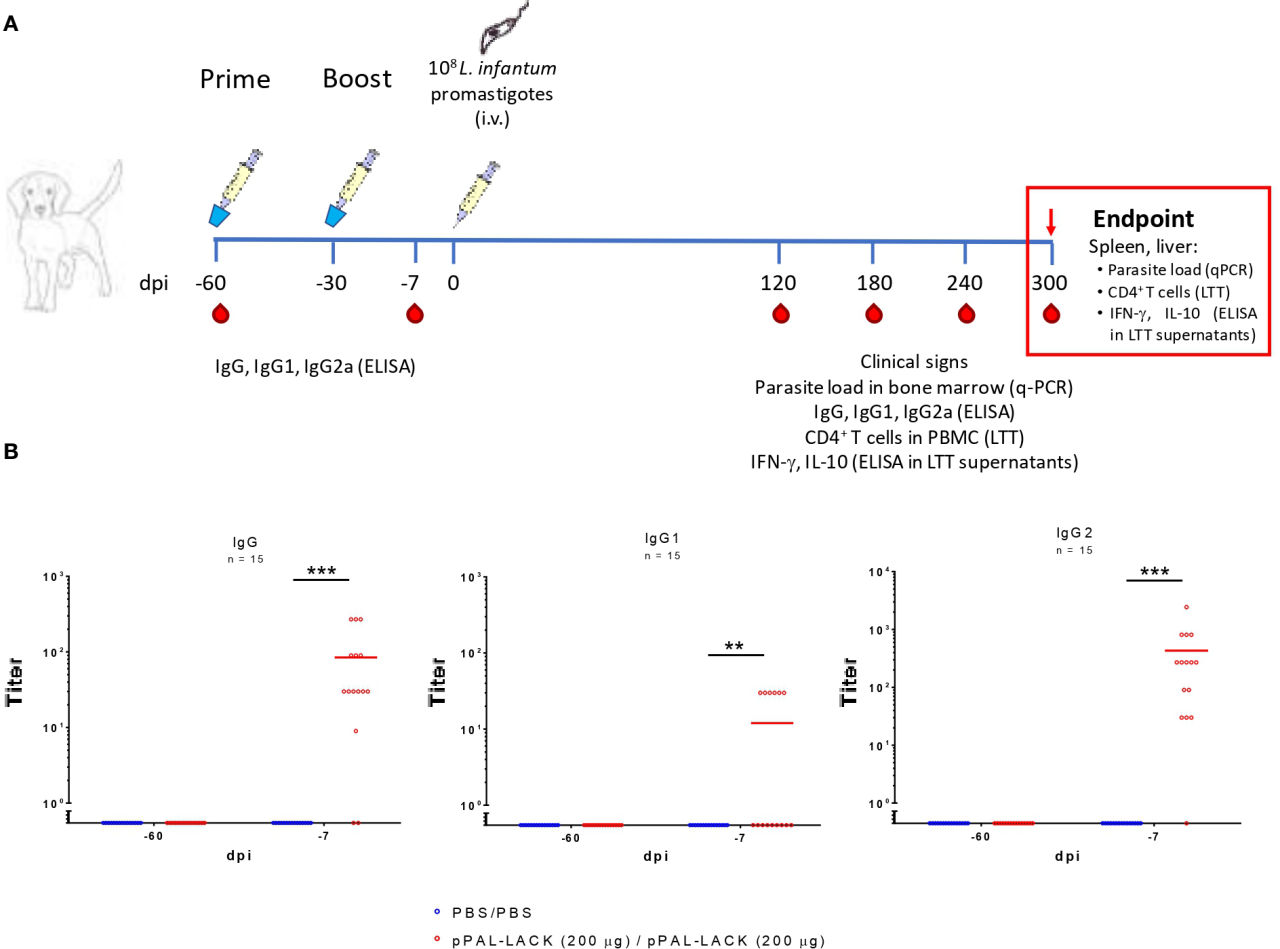
The pPAL-LACK vaccination schedule and pre-challenge circulating IgG levels. **(A)** Timeline of the preclinical efficacy experiment in Beagle dogs. The vaccinated group received two doses (prime/boost, -60 and -30 dpi) of 200 μg pPAL-LACK in 1 mL of sterile PBS and the infection control group received 1 mL of sterile PBS by the intranasal route. Both groups were challenged with 10^8^ *L. infantum* promastigotes by the intravenous route. Red drops indicate the date of blood sampling. **(B)** Pre-challenge circulating IgG, IgG1, and IgG2 anti-SLA titers (-60, -7 dpi). Blue dots represent unvaccinated animals. Red dots represent vaccinated animals. Statistical inference was performed using the Chi-square test (**p<0.01, ***p<0.001).

The preclinical efficacy trial of the DNA vaccine followed the EU’s and Spain’s animal experimentation ethics regulations. To conduct the experiment properly, all measures available were taken to ameliorate the suffering of thirty ~13 kg, 12 to 18-month-old Beagle dogs (CEDS, France). The dogs were lodged in groups of five in 18 m^2^ kennels and fed *ad libitum*. The animals were maintained in optimal conditions: temperature control, disease monitoring, behavioral enrichment, daily controlled release outdoors, etc. The experimental procedures were performed under 20 mg/kg medetomidine hydrochloride anesthesia and reversed with 100 mg/kg atipamezole hydrochloride. Experimental design and procedures were approved by the University of Zaragoza Ethics Advisory Commission for Animal Experimentation (11/03/2010, reference PI12/10). Two animal groups were established: i) positive infection control or placebo group (15 animals) inoculated with sterile endotoxin-free PBS twice before challenge; and ii) vaccinated group (15 animals) inoculated with two doses (prime/boost) of 200 μg pPAL-LACK dissolved in sterile endotoxin-free PBS (1 mL final volume). This plasmid dose is double of what was specified by Ramos et al. (18, 19) to obtain an improved efficacy of the homologous plasmid-plasmid regimen and to avoid the usage of a vaccinia recombinant virus. The experiment schedule is described in **Figure 1A**. The prime and boost inoculations were performed by the intranasal route 60 and 30 days before challenge (-60 and -30 dpi), respectively. The infectious challenge consisted of 10^8^ highly infective stationary phase *L. infantum* promastigotes. This experimental infection was performed 30 days after the booster dose (0 dpi) by the intravenous route. 2 mL blood samples were taken 60 days and 7 days before the infectious challenge (-60 dpi and -7 dpi) for humoral response analysis. 10 mL blood samples were taken at 120, 180, 240, and 300 dpi for humoral and cellular immune response analysis. 100 μL bone marrow samples were obtained at 0, 120, 180, and 240 dpi for parasite burden analysis. At the end of the study (300 dpi), dogs were euthanized using 0.3 mL/kg T61 (Intervet-MSD Animal Health, Madrid, Spain) by the intravenous route. Liver, lymph nodes, bone marrow, and spleen samples were taken during the necropsy for cellular immune response analysis.

### Evaluation of clinical signs

Canine leishmaniasis clinical signs (anemic mucosa, epistaxis, conjunctivitis, lymphadenomegaly, skin lesions such as exfoliative dermatitis, ulcers, etc.) were registered in clinical record forms on days 120, 180, 240, and 300 dpi. The intensity of each clinical sign was scored on a natural number scale ranging from 0 to 4. The clinical score for a given animal is the sum of all scores registered. Clinical signs were obtained by two independent evaluators.

### Parasite burden evaluation

Genomic DNA was isolated from bone marrow aspirates using NucleoSpin^®^ Blood (Macherey-Nagel, Düren, Germany) following the manufacturer’s instructions. Parasite load evaluation by qPCR was performed with the LEISH-1 primer (AACTTTTCTGGTCCTCCGGGTAG), LEISH-2 primer (ACCCCCAGTTTCCCGCC), and LEISH-P TaqMan MGB probe (6-FAM-AAAAATGGGTGCAGAAAT) originally designed by Francino et al. (40) for minicircle detection and still widely used (14, 41–44).

### Humoral immune response assessment

2 mL peripheral blood collected from dogs was left to clot at room temperature for 20 min using Eurotubo^®^ Serum Separation polypropylene tubes (Deltalab, Barcelona, Spain) with accelerant and granules, and centrifuged at 10,000x*g* at room temperature for 10 min. Serum was stored at -20°C and used for ELISA assessment of total IgG, IgG1, and IgG2 against Soluble *Leishmania* Antigen (SLA) levels. Circulating IgGs were titrated by ELISA using HRP-conjugated protein A and anti-dog IgG1 and IgG2, as described (14).

### T-cell response evaluation

The lymphoblastic transformation test (LTT) was performed on peripheral blood mononuclear cell (PBMC) samples and in target organs (liver, lymph node, and spleen) with LACK and CLA following a procedure previously described (14). The IL-10 and IFN-γ concentrations were evaluated in LTT supernatants. This was carried out using the canine IL-10 and IFN-γ DuoSet^®^ ELISA Development System kits (R&D Systems, Minneapolis, MN.) according to the manufacturer’s instructions. We used IL-10 instead of IL-4 as a marker for the Th2 response because previous experiments indicate that IL-4 data in canine leishmaniasis are variable and unclear (19, 45, 46).

### Statistical analysis

The differences in antibody titers between the control and the vaccinated group were analyzed using the Chi-square test because both the independent and the dependent variables are categorical. According to the outcomes of the Kolmogorov-Smirnov normality test and the Levene homoscedasticity test (**Table S1**), ANOVA was applied to clinical signs, and the Mann-Whitney U (α= 0.05) test was applied to parasite burden and cellular immune response data.

## Results

### Specific anti-SLA antibodies after prime/boost immunization of Beagle dogs with the pPAL-LACK plasmid

Circulating IgG levels in vaccinated dogs were titrated against SLA before challenge to determine the presence of specific antibodies against *Leishmania* antigens (**Figure 1B**). Considering the cutoff level equal to twice the background value, the total IgG titers ranged between 30 and 270 in vaccinated dogs. The vaccination regimen induced low circulating IgG1 production. Only six dogs presented circulating IgG1 titers equal to 30. The IgG2/IgG1 ratio is ∼35. The IgG2 levels account for most IgG production. As expected, none of the unvaccinated dogs produced specific antibodies. Therefore, inoculation with the DNA plasmid containing the LACK protein’s encoding gene induces a specific humoral response against SLA extracts mainly based on IgG2 antibodies, suggesting Th1 cell subset activation.

### Clinical signs and parasite burden

**Figure 2:**
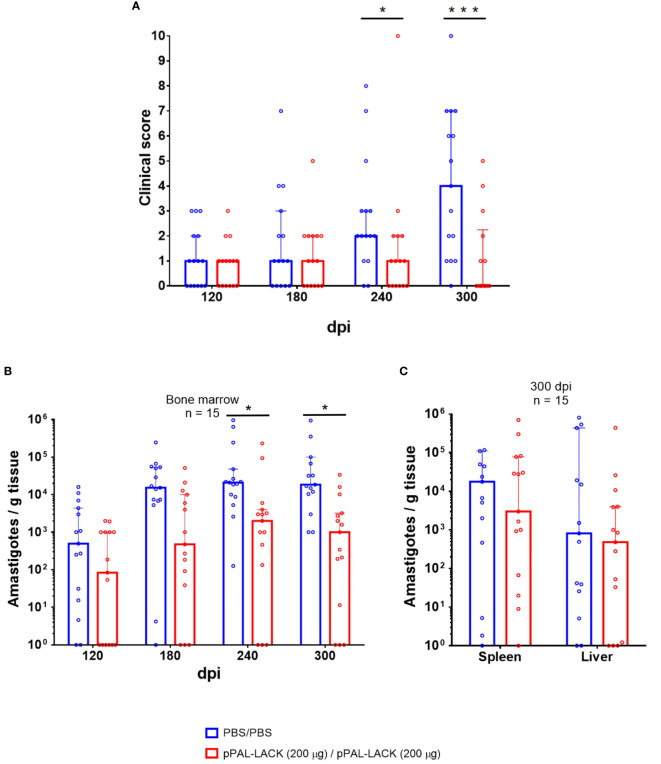
Clinical signs and parasite burden. **(A)** Clinical score. The clinical signs of vaccinated and control Beagle dogs 120, 180, 240, and 300 dpi are represented. Values are the sum of 0-4 scores provided for each of the clinical signs observed. The following clinical signs (anemic mucosa, epistaxis, conjunctivitis, lymphadenomegaly, skin lesions such as exfoliative dermatitis, ulcers, etc.) were evaluated by two independent persons. Positive standard deviation value bars have been represented. Two dogs had to be euthanized before the end of the experiment (after the 240 dpi time point) for ethical reasons. ANOVA was performed for statistical inference of differences in the clinical score between both groups (*p<0.05, ***p<0.001). **(B)** Parasite burden in bone marrow (120, 180, 240, and 300 dpi). Bone marrow biopsy was obtained by aspirate from the animals under anesthesia. **(C)** Endpoint parasite burden in the spleen and liver (300 dpi). Genome DNA was extracted from tissue samples. The parasite burden was evaluated by qPCR. The number of amastigotes per gram of tissue in a logarithmic scale is represented. Statistical inference was performed with the Mann-Whitney U test (*p<0.05).

No clinical signs were developed until 120 dpi in both animal groups. The clinical profiles of the control and the vaccinated groups were similar at 120 and 180 dpi. At 240 dpi, the average clinical score was approximately double in the unvaccinated controls than in the vaccinated group. At the experiment’s endpoint, the clinical signs subsided in 60% of vaccinated dogs and the rest displayed signs to a lesser extent. The control group’s clinical profile worsened in most dogs (**Figure 2A**). Therefore, homologous prime/boost vaccination of dogs with 200 µg/dose pPAL-LACK leads to a progressive decrease in clinical signs post-challenge in vaccinated animals towards the end of the experiment, once the disease is clearly established. At this time point, nine animals (60%) display a complete lack of clinical signs (clinical score 0), three animals present mild signs (mean clinical score 1), and only three animals show medium clinical signs (mean clinical score 4) (**Figure 2A**).

After successful infection of the control and vaccinated groups with 10^8^ *L. infantum* infective promastigotes, a statistically significant reduction in parasite burden was observed in vaccinated dogs over time (Mann-Whitney U test, α = 0.05). The average amastigote number per gram of bone marrow was lower (1-2 orders of magnitude) in the vaccinated group throughout the experiment (120, 180, 240, and 300 dpi) (**Figure 2B**). A reduction of approximately 92% in parasite load was registered in this tissue at 300 dpi as observed in the logarithmic scale in **Figure 2B**, which is statistically significant according to the Mann-Whitney U test (α = 0.05). The parasite load in spleen and liver is also clearly reduced (88% and 42%, respectively) (**Figure 2C**).

### Humoral immune response after challenge

**Figure 3:**
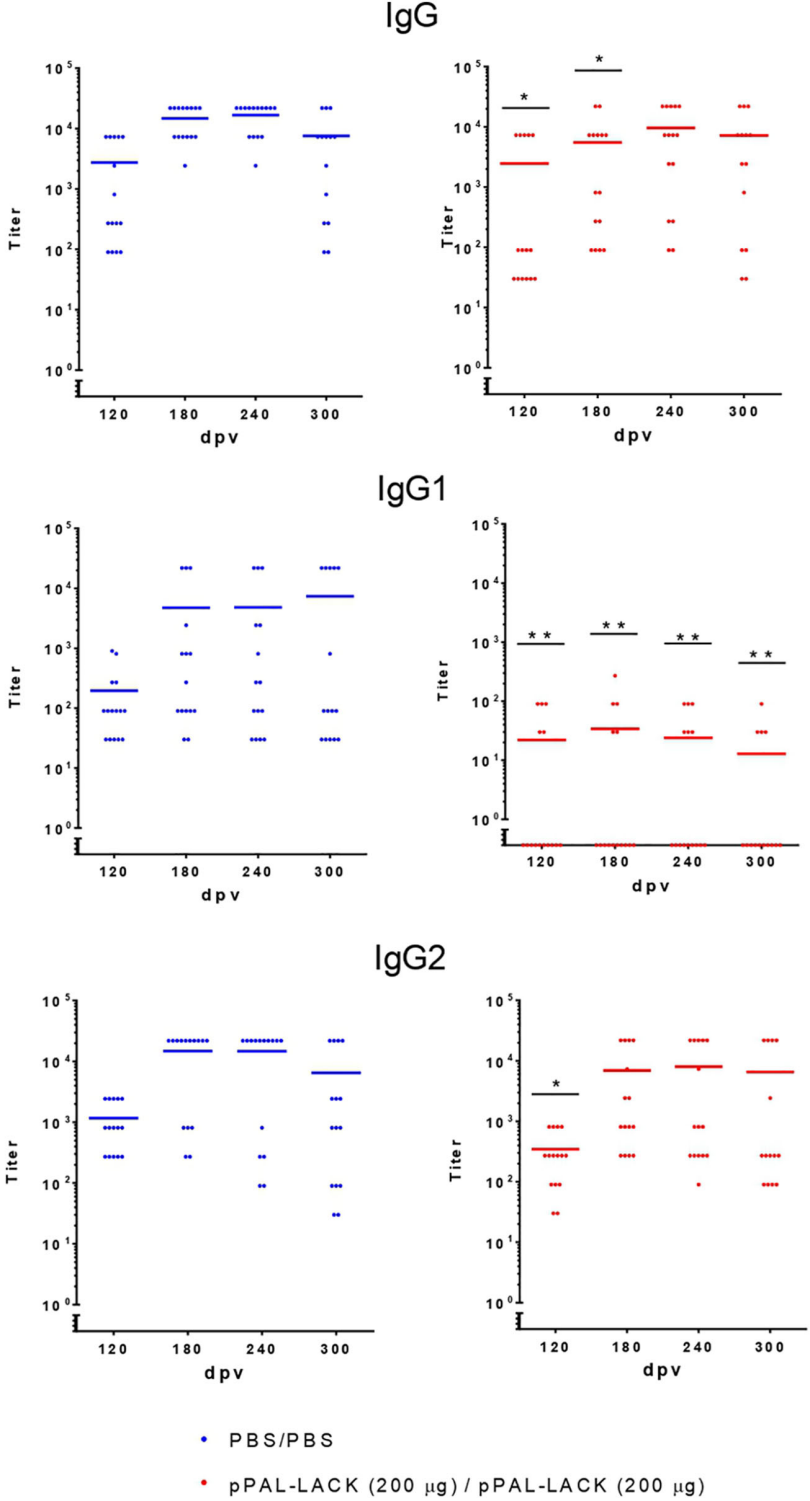
Circulating *L. infantum*-specific IgG levels after challenge. Circulating IgG, IgG1, and IgG2 anti-SLA titers throughout the experiment. Blue dots: unvaccinated infected dogs. Red dots: vaccinated infected dogs. Statistical inference was performed using the Chi-square test (*p<0.05, and **p<0.01).

As shown in **Figure 3**, serology was positive against SLA for all control and vaccinated dogs after the infectious challenge according to the total levels of specific circulating IgGs, indicating activation of the humoral response. The total IgG titers were similar in vaccinated and unvaccinated dogs, indicating that infection was successful in both groups. However, IgG1 and IgG2 subclasses displayed clear differences between groups. Whereas the control dogs showed high IgG1 and IgG2 titers, the vaccinated dogs specifically showed high IgG2 titers and very low IgG1 titers (see **Figure 3**). Therefore, the IgG2/IgG1 ratio was considerably higher in the vaccinated group compared to the control group (the quotients equal ∼200 and ∼3, respectively). These results suggest a T-cell response skewed towards the Th1 subset in protected dogs.

### Cellular immune response after challenge

**Figure 4:**
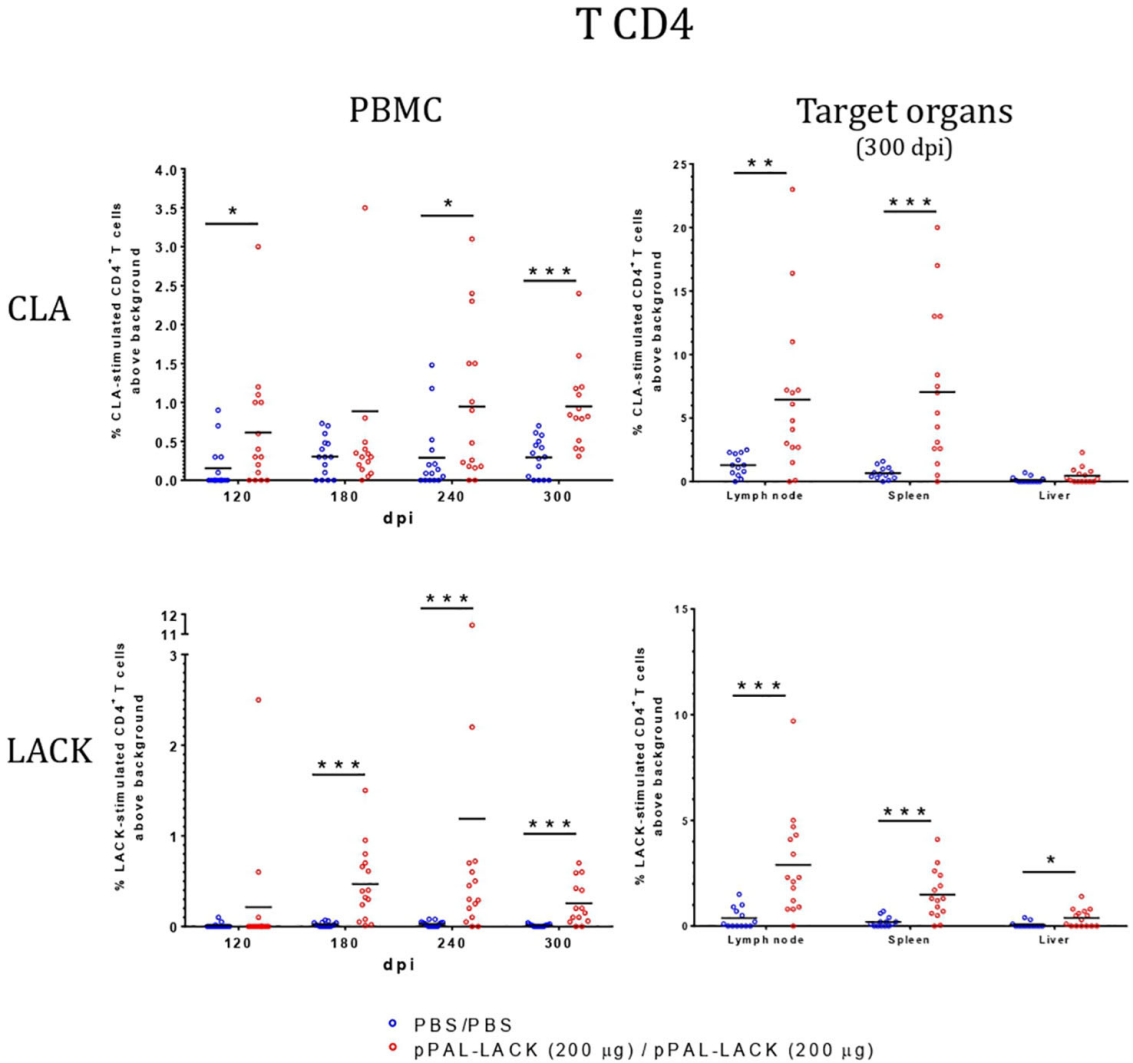
Lymphoblastic transformation test (LTT). CD4^+^-proliferation against CLA is higher in vaccinated dogs in PBMC and target organs. Blue dots: unvaccinated infected dogs. Red dots: vaccinated infected dogs. Statistical inference was performed with the Mann-Whitney U test (*p<0.05, **p<0.01, ***p<0.001).

**Figure 5:**
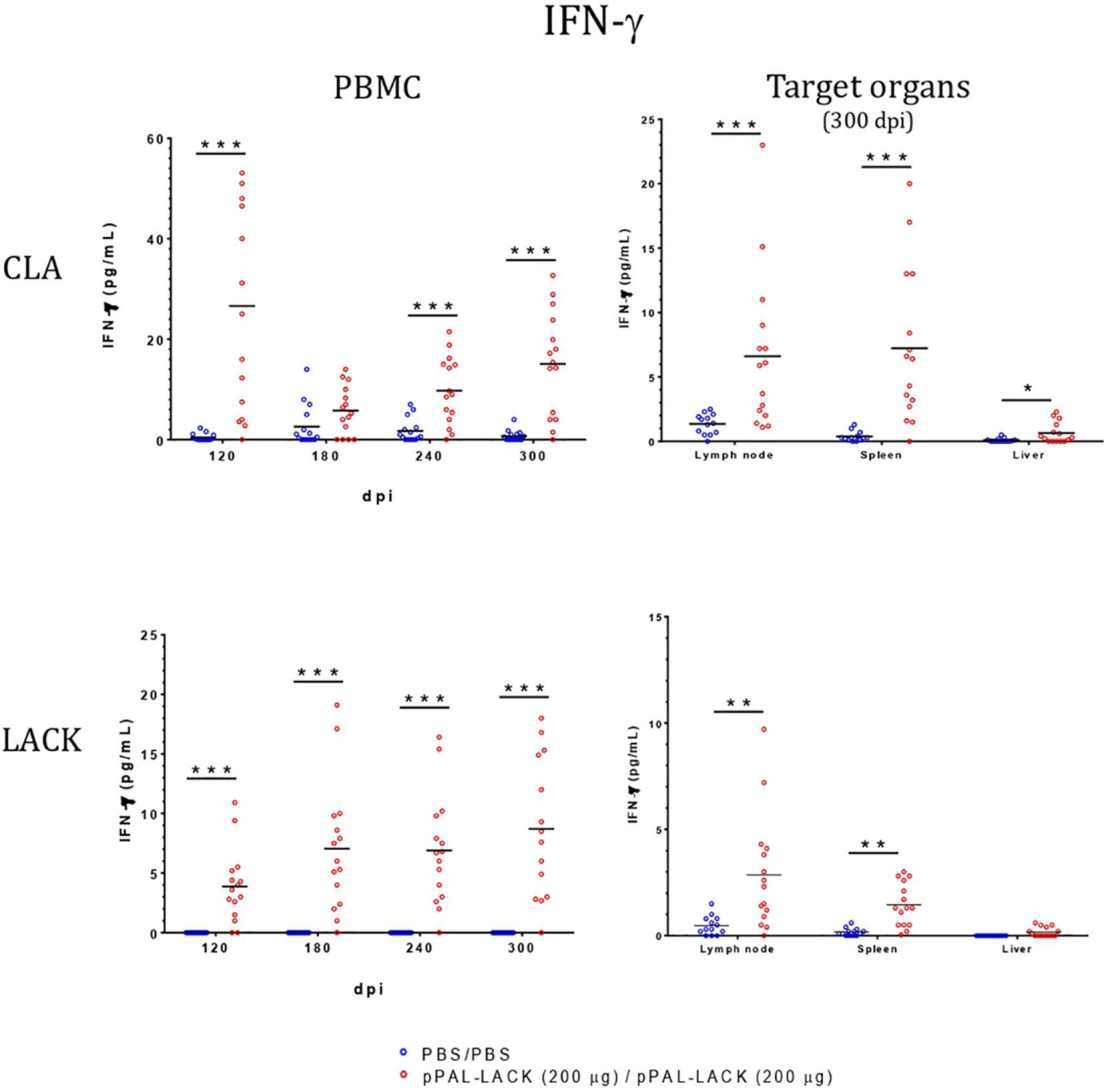
IFN-γ concentration in LTT supernatants. A specific commercially available ELISA procedure was used to determine IFN-γ concentration (pg/mL) in LTT supernatants, which were immediately stored at -20°C after separation from cells. Blue dots: unvaccinated infected dogs. Red dots: vaccinated infected dogs. Statistical inference was performed with the Mann-Whitney U test (*p<0.05, **p<0.01, ***p<0.001).

**Figure 6:**
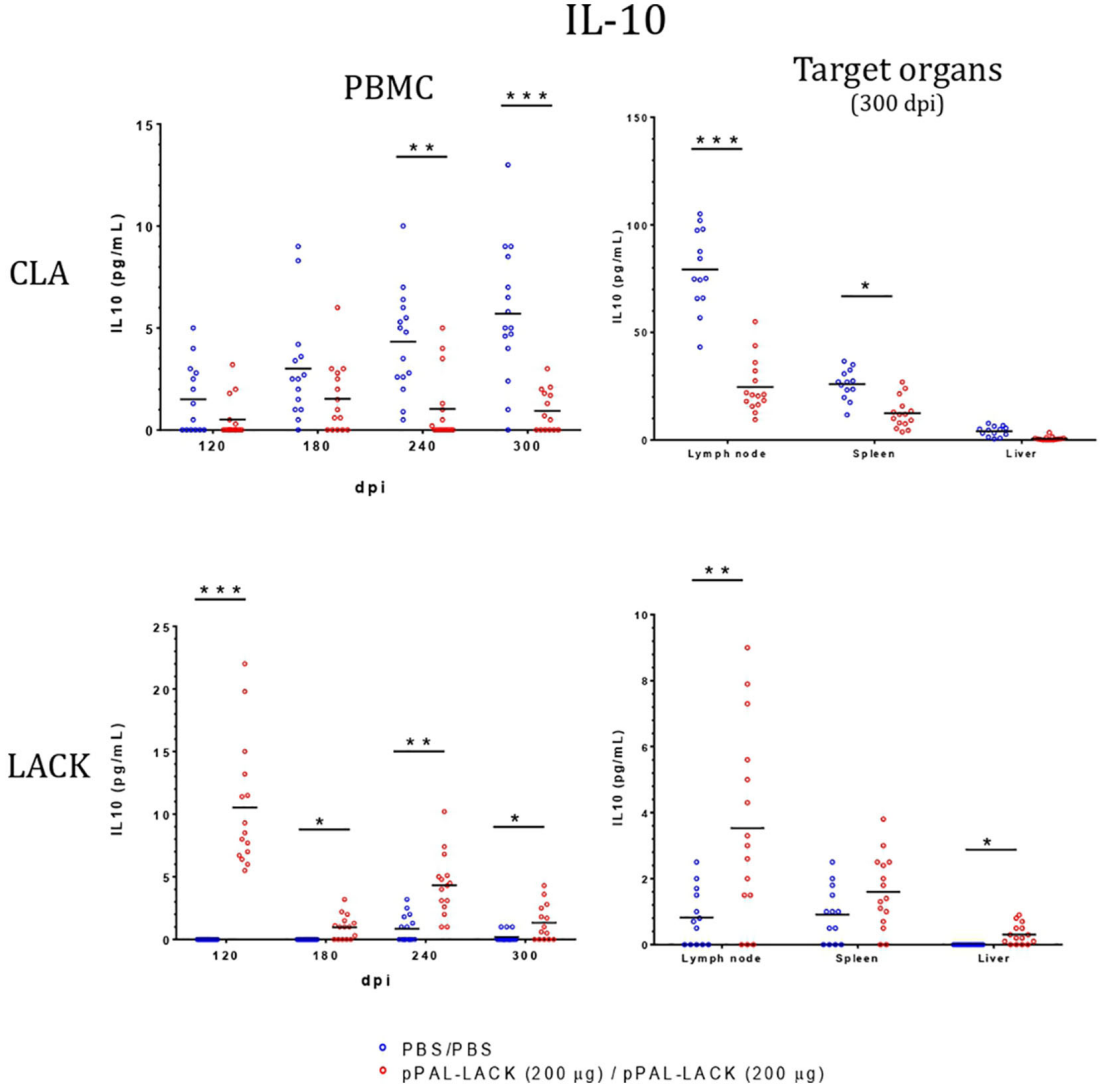
IL-10 levels in LTT supernatants. A specific commercially available ELISA procedure was used to determine IL-10 concentration (pg/mL) in LTT supernatants, which were immediately stored at -20 °C after separation from cells. Blue dots: unvaccinated infected dogs. Red dots: vaccinated infected dogs. Statistical inference was performed with the Mann-Whitney U test (*p<0.05, **p<0.01, ***p<0.001).

LTT experiments on CD4^+^ cells obtained from PBMC revealed that the average proliferation rate against CLA is higher in all vaccinated animals than in control animals at all experiment time points (**Figure 4**). There is also more proliferation in lymph nodes and spleen cells from vaccinated dogs compared to controls at the end of the experiment. No cell activation was observed in the liver. The protective antigen LACK induced proliferation in all CD4^+^ T cell populations analyzed as expected. The differences between vaccinated and control animals are statistically significant at all times except for 180 dpi against CLA and 120 dpi against LACK (Mann-Whitney U test, α = 0.05). The secreted IFN-γ and IL-10 concentrations in the LTT supernatants account for the Th1 and Th2 immune responses, respectively. The average IFN-γ levels secreted by CD4^+^ T cells from PBMC against CLA were high at all times with respect to control animals. The same is observed in the target organs (**Figure 5**). PBMC, lymph node cells, and splenocytes produced a high amount of IL-10 in unvaccinated animals compared to the vaccinated group. Again, Th1 activation is observed in target organs of vaccinated dogs, except for the liver (Mann-Whitney U test, α = 0.05) (**Figure 6**). Activation by the LACK antigen was also observed. Both cytokines were produced due to the specificity of the elicited response.

## Discussion

The development of vaccines designed to prevent parasitic infections has many hurdles. This is due to complex parasite-host interactions, intricate parasite life cycles, parasite evasion from the host’s immune system, and other complex mechanisms of pathogenicity not yet characterized. A remarkable example of these difficulties is the RTS,S/AS01 vaccine against malaria, which is an outstanding milestone achieved against this particularly complex disease. This vaccine has been recommended by the WHO even though it only confers 39% protection against malaria up to 4 years and 30% up to 10 years (47–51).

Several vaccines of different types against ZVL have been developed in the last 30 years. However, most are not available anymore (52, 53). One of them was a first-generation vaccine composed of attenuated parasites. A field trial of this vaccine in Iran was completed (54) but no information on distribution has been made available since then. In recent works (55, 56), authors claim to have halted the appearance of clinical signs for one year. However, this type of vaccine is based on the use of knockout parasites whose long-term stability and security are not guaranteed. Two ZVL vaccines were developed in Brazil: Leishmune^®^, which is a second-generation vaccine based on gp63 *Leishmania* surface glycoprotein (57) and was discontinued in Phase III by the Brazilian government (52, 53), and Leish-Tec^®^, a third-generation vaccine based on the recombinant A2 antigen gene cloned in a recombinant adenovirus vector, which delayed the manifestation of clinical signs with respect to controls. However, the parasite was later detected in four out of seven test dogs (52, 53). Additionally, the vaccine causes several side effects (58) and no field result has been published since then. Of the two vaccines developed in Europe, one known as Canileish^®^ is based on *Leishmania* secreted proteins and was approved in 2011. However, antibodies induced by this vaccine obscure disease diagnosis (59) and recent studies using insecticide collars did not find significant differences between vaccinated and unvaccinated dogs used in the study (52). The last vaccine to be made available in the European market against ZVL is Letifend^®^, licensed in 2016. It is based on the fusion of five different epitopes from histones and ribosomal proteins (60) and induces about 70% protection in clinical signs. However, no data on parasite burden reduction was provided (WorldLeish7 Congress, Cartagena de Indias, Colombia, 2022) and its value to control epidemiological transmission is unknown. Therefore, new vaccines providing safer and more reliable protection are required. Our DNA vaccine provides a protection rate of at least 60% with respect to the clinical signs, plus a remarkable reduction in the number of parasites present in the bone marrow (92%), spleen (82%), and liver (42%), which suggests a lower rate of disease transmission in vaccinated dogs, which fits with the One Health objectives.

DNA vaccines are a promising method of control. They are safe and can be easily modified to include other protective genes, facilitating the development of bivalent or multiple vaccines to achieve better protection against infectious agents that efficiently evade the host’s immune system. Their manufacturing process is low-cost and relatively simple and their thermotolerance during limited periods would facilitate worldwide distribution (61).

This study encompasses the preclinical phase of development of the pPAL-LACK vaccine against canine leishmaniasis. The dog model cannot be replaced by conventional laboratory animal models because it is the main natural reservoir of ZVL, and the behavior of the parasite-host interaction in the widely studied mouse model is different from the mechanisms previously described. Even though dog experimentation is complex, it is essential for ZVL vaccine development. In addition to host genetic variability, there are differences in disease manifestation between the dog and mouse models that might be considered. However, T-cell proliferation, which is essential for protection against *L. infantum* infection, displays similarities between the dog and mouse models (12, 17, 20, 21). The protective response skewed towards Th1 proliferation is very clear among vaccinated animals in the advanced phases of infection.

Previous results from our laboratory showed that the LACK antigen gene administered in a mammalian expression plasmid vector confers partial protection in mice against *L. major* (32, 33). Other experiments demonstrated that protection was adequate when using a heterologous regimen that included the recombinant plasmid in the prime dose and a recombinant modified vaccinia virus containing the LACK gene [MVA-LACK (Ankara strain)] in the booster dose inoculated subcutaneously (18, 19, 35). Intranasal delivery following the plasmid/plasmid homologous regimen elicited significantly more elevated protection than the subcutaneous route (32). Therefore, the pPAL-LACK plasmid can be considered a more effective and safer vaccination vehicle (15, 16). Additionally, the plasmid carrying the LACK gene contains CpG islands as a molecular adjuvant and was shown to be more effective when using the intranasal route (15, 16). Unexpectedly, the addition of plasmids containing the encoding genes of canine IL-12 p35 and p40 subunits, supposedly favoring activation of the Th1 subset, did not improve the level of protection acquired by pPAL-LACK vaccination (14). Therefore, these plasmids were not included in the final vaccine formulation. These data also suggest the existence of a finely tuned regulatory mechanism of response to infection.

The LACK gene-based canine leishmaniasis vaccine trials with a different inoculation route and different vaccination regimens have shown notable but partial protection against challenge with a high number (10^8^) of *L. infantum* infective promastigotes (18, 19, 35). The pPAL-LACK vaccine formulation against canine leishmaniasis consists of a sterile endotoxin-free PBS solution containing the pPAL-LACK construct administered in two doses by the intranasal route, which effectively protects Beagle dogs from challenge (**Figure 2**). The infectious challenge was successful in all dogs according to *L. infantum* load analysis by qPCR and titration of circulating *Leishmania*-specific IgG. Sixty percent of vaccinated dogs did not show any clinical signs 300 dpi and all other vaccinated dogs showed moderate clinical signs. The average parasite burden in bone marrow was up to 92% lower in vaccinated dogs compared to control dogs. The spleen and liver also showed reduced parasite load, while the infection was fully established in control animals (**Figure 2**). Therefore, pPAL-LACK significantly reduces the characteristic presence of the parasite in the bone marrow and other target organs, a key feature of the ZVL chronic disease.

In mammals, *L. infantum* is an intracellular parasite. Thus, the presence of circulating antibodies does not guarantee protection. High levels of IgG production do not imply effectiveness in infection control. Antibody production indicates a response against the parasite by the host´s immune system and the type of antibodies reveal the kind of response: lower levels of IgG2 are usually associated with disease progression and high levels of IgG2 are associated with cellular activation, which is required for the control of infection. The pPAL-LACK plasmid induces a specific IgG2 humoral response (**Figures 1B**, **3**) with similar total IgG titers in both groups. However, the IgG2/IgG1 ratio is much higher in vaccinated dogs, which is likely related with the induction of a Th1 immune response, essential for the clearance of the parasitic infection. This is confirmed in LTT experiments measuring the cellular response, which show that a Th1 response is elicited in vaccinated dogs against CLA extracts and the LACK antigen after *L. infantum* challenge. Hence, this trial with two groups of 15 animals confirms that the LACK gene protects against canine leishmaniasis with a homologous plasmid/plasmid formulation. The protection levels reached with homologous prime/boost intranasal vaccination are similar to the protection levels achieved by the subcutaneous route using the previously tested heterologous system that includes a recombinant virus (18, 19, 35).

CLA activation in PBMC showed IFN-γ levels that were higher in the vaccinated dogs with respect to control dogs throughout the experiment, as observed in previous experiments using other vaccination routes (15, 18), indicating the specific activation of the immune system that results in disease control. There is no IFN-γ production in control dogs. The IL-10 levels secreted by CLA-stimulated PBMC are lower at all time points in vaccinated dogs. On the contrary, IL-10 values were higher in the unvaccinated animals compared to those protected. At the endpoint, target organ IFN-γ values were higher in the vaccinated group, whereas the IL-10 levels were higher in the unvaccinated group (**Figure 6**). IFN-γ production increased in response to the LACK antigen throughout the experiment in all vaccinated dogs and were higher than those registered in the controls. This suggests that the DNA vaccine induces specific immune system activation.

Taken on the whole, the data suggest that the DNA plasmid, pPAL-LACK, which contains the encoding gene of the protective LACK antigen, inoculated by the intranasal route, elicits a robust humoral and cellular immune response skewed towards Th1 and, therefore, protects against an elevated number of highly infective *L. infantum* promastigotes. This vaccine is safe and confers protection against *L. infantum* in approximately 60% of Beagle dogs in terms of complete clearance of clinical signs. The average reduction of the parasite burden is ∼92% in bone marrow, ∼82% in the spleen, and ∼42% in the liver at the experiment endpoint. This reduction is statistically significant (Mann-Whitney U test, p<0.05).

This effective DNA vaccine against canine leishmaniasis displays several advantages over existing vaccines based on recombinant proteins (62). It induces a Th1 cellular response, promoting protection from the parasite, and is thermostable. Consequently, the cold chain is not required, which facilitates distribution to dogs in shelters, to hunting dogs, and in developing countries. The formulation is simple and includes CpG islands as a molecular adjuvant and no other conventional adjuvant. Intranasal delivery with a nebulizer ensures no loss of vaccine volume.

In summary, homologous prime/boost inoculation by the intranasal route of the non-replicative, antibiotic resistance-free DNA vaccine against ZVL containing the encoding *L. infantum* LACK gene leads to high protection of vaccinated dogs compared to controls. This includes a decrease in disease clinical signs plus a decrease of over 90% in parasite burden.

## Data availability statement

The original contributions presented in the study are included in the article/**Supplementary Material**. Further inquiries can be directed to the corresponding author.

## Ethics statement

The animal study was approved by University of Zaragoza Ethics Advisory Commission for Animal Experimentation (11/03/2010,PI12/10). The study was conducted in accordance with the local legislation and institutional requirements.

## Author contributions

VL and JC conceived the study. VL, JC, AA, and PA designed the study. VL and JC obtained funding. AA, PA, JL, MPP, AE, AC, and SR-G performed the experiments. AA and PA performed analysis and prepared the figures. VL wrote the manuscript. VL, PA, and AA edited the manuscript. All authors contributed to the article and approved the submitted version.
